# The Cross-Sectional Area Assessment of Pelvic Muscles Using the MRI Manual Segmentation among Patients with Low Back Pain and Healthy Subjects

**DOI:** 10.3390/jimaging9080155

**Published:** 2023-07-31

**Authors:** Wiktoria Frącz, Jakub Matuska, Jarosław Szyszka, Paweł Dobrakowski, Wiktoria Szopka, Elżbieta Skorupska

**Affiliations:** 1 Faculty of Biomedical Sciences, Medical University of Lodz, Al. Kosciuszki 4, 90-419 Lodz, Poland; wiktoria.fracz@stud.umed.lodz.pl; 2 Department of Physiotherapy, Poznan University of Medical Sciences, ul. 28 czerwca 1956r. nr 135/147, 61-545 Poznan, Poland; jakub.matuska@student.ump.edu.pl; 3 Doctoral School, Poznan University of Medical Sciences, Bukowska 70, 60-812 Poznań, Poland; 4 Doctoral School, Rovira I Virgili University, Carrer St. Llorenç No. 21, 43201 Reus, Spain; 5 Opole Rehabilitation Centre in Korfantów, Wyzwolenia 11, 48-317 Korfantów, Poland; 6 Psychology Institute, Humanitas University in Sosnowiec, 41-200 Sosnowiec, Poland; 7 Faculty of Veterinary Medicine and Animal Science, Poznan University of Life Sciences, 60-637 Poznań, Poland

**Keywords:** sciatica, muscle atrophy, inter-rater reliability, manual segmentation, magnetic resonance imaging

## Abstract

The pain pathomechanism of chronic low back pain (LBP) is complex and the available diagnostic methods are insufficient. Patients present morphological changes in volume and cross-sectional area (CSA) of lumbosacral region. The main objective of this study was to assess if CSA measurements of pelvic muscle will indicate muscle atrophy between asymptomatic and symptomatic sides in chronic LBP patients, as well as between right and left sides in healthy volunteers. In addition, inter-rater reliability for CSA measurements was examined. The study involved 71 chronic LBP patients and 29 healthy volunteers. The CSA of gluteus maximus, medius, minimus and piriformis were measured using the MRI manual segmentation method. Muscle atrophy was confirmed in gluteus maximus, gluteus minimus and piriformis muscle for over 50% of chronic LBP patients (*p* < 0.05). Gluteus medius showed atrophy in patients with left side pain occurrence (*p* < 0.001). Muscle atrophy occurred on the symptomatic side for all inspected muscles, except gluteus maximus in rater one assessment. The reliability of CSA measurements between raters calculated using CCC and ICC presented great inter-rater reproducibility for each muscle both in patients and healthy volunteers (*p* < 0.95). Therefore, there is the possibility of using CSA assessment in the diagnosis of patients with symptoms of chronic LBP.

## 1. Introduction

Low back pain (LBP) affects approximately 7.5% of the global population [1]. Sciatica is diagnosed in 23% to 57% LBP patients [2]. Epidemiological data confirm that 10% to 40% of the world’s population will experience sciatic symptoms at least once in a lifetime [3]. The annual incidence rate is determined to be 1 to 5% of the adult population, with the occurrences peaking in the fourth decade of life [3].

It was confirmed that in about 30% of patients with chronic low back pain, symptoms persist for longer than a year [4]. Neuropathic or nociplastic pain states are the main mechanisms for chronic state of pain among sciatica patients. Importantly, these mechanisms may coexist, as is the case with low back pain, including sciatica [5]. According to the World Health Organization’s guidelines, a new approach in the treatment of chronic pain is necessary to provide adequate therapy to the leading pain pathomechanism [6,7] Currently, there are no objective methods to determine the main chronic pain pathomechanism in individual patients [8,9]. It is recommended to explore the new diagnostic methods to identify subpopulations with specific clinical characteristics as a guide to changes in treatment strategies [6].

Magnetic resonance imaging (MRI) is a standard method in the diagnosis of patients with symptoms of LBP but constitutes rather unproductive in patients with chronic low back pain with the exception of muscle segmentation of acquired resonance images [10,11,12,13,14]. Patients with chronic LBP present morphological changes, such as alterations in muscle activity and strength associated with variations in volume and cross-sectional area (CSA) of the lumbopelvic region. Degenerative changes in the muscles of this region presenting among patients with chronic lumbar pain conditions are characterized by: decreased total CSA and functional CSA values (representing the non-adipose muscle mavalues), increased adipose component in certain muscles (fat infiltrations and deposits), changes in muscle density, and changes in volume of muscle tissue. The reduced activity caused by these morphological changes (e.g., muscle atrophy) calculated based on CSA and muscle volume may serve as an indicator of chronic LBP and can be analyzed by MRI segmentation. Additionally, a modern approach of diagnosing LBP highlights the value of muscle tissue assessment as an additional aspect that potentially facilitates pain diagnosis in this area [13,14,15,16]. There are several studies of successful multifidus muscle segmentation showing muscle atrophy in patients presenting symptoms of chronic low back patients [17,18,19,20,21,22]. Moreover, statistically significant changes in muscle volume of the pelvic muscles (gluteus maximus, medius and minimus, as well as the piriformis muscle) were confirmed on the symptomatic side in patients with chronic LBP with the use of manual segmentation technique [11]. Manual segmentation, which is considered the “gold standard” in medical image segmentation, allows the most precise analysis of magnetic resonance images. Moreover, cross-sectional area parameter can be obtained faster than volumetric measurement, which is especially important in time consuming manual segmentation [3,10,12,14,23].

Therefore, the main aim of the study was to assess whether CSA measurements of the gluteal muscles (minor, medius, major) and the piriformis muscle will indicate changes in the morphometric parameters, to detect signs of muscle atrophy between the asymptomatic and symptomatic sides in patients with chronic LBP, as well as between the right and left sides in healthy individuals. Moreover, the inter-rater reliability of manual segmentation for CSA measurements of those muscles in patients with chronic low back pain and healthy volunteers was examined.

## 2. Materials and Methods

The study was approved by the Ethics Committee of the Poznan University of Medical Sciences (resolution number 50/23). Before data collection, written informed consent was obtained from all participants.

### 2.1. Participants

An amount of 71 magnetic resonance images were assessed for eligibility. The MRI images were obtained involving patients with chronic low back pain (37 men, 34 women; mean age 43.21 ± 8.43) and healthy volunteers (14 men, 15 women; mean age 44.35 ± 7.91). The clinical data of chronic LBP patients who underwent the diagnostic procedure before recruitment to the rehabilitation clinic with/without history of rehabilitation process were analyzed. Table 1 shows a clinical description of all participants.

The inclusion criteria for chronic low back pain patients were as follows: chronic pain in the lumbosacral area for more than 3 months, recurrent stay, leg pain scores > 3 on the Visual Analogue Scale (VAS) and pain in the examined area as the dominant pain condition, positive Lasègue’s sign, age between 30 and 60 years, current MRI examinations, the presence of both lower limbs. The inclusion criteria for healthy volunteers were as follows: absence of chronic low back pain history, general good health, age between 30 and 60 years, current MRI examinations, the presence of both lower limbs. The exclusion criteria for all participants were as follows: history of spinal surgery, tumors in the examined area, diabetes, scoliosis, epilepsy, infection, pregnancy, complex regional pain syndrome, cauda equina syndrome, inflammatory rheumatologic diseases, history of stroke, previous back surgery, spinal tumors, history of oncological treatment, coagulation treatment and disseminated intravascular coagulation.

### 2.2. Magnetic Resonance Imaging: Image Acquisition

Magnetic resonance imaging of the lumbosacral spine was performed in qualified patients with chronic low back pain and healthy volunteers. Medical images were obtained using a Tesla Signa HDe system scanner with 1.5 T power (GE HealthCare, Korfantów, Poland). Data acquisition covered the area from the upper thigh to the pelvis and lumbar spine bilaterally. During data acquisition, patients were placed in supine position. T2 sagittal images were acquired with pulse sequence with the following parameters: echo time (TE): 110, repetition time (TR): 3.500, field of view (FOV): 100%, matrix: 320 × 224, slice thickness of 4 mm. The images were exported to the DICOM format and then converted to the NIfTI format enabling their segmentation in the ITK-SNAP program.

### 2.3. Data Analysis: Image Segmentation

Manual segmentation technique, which is considered the “gold standard” in medical image segmentation and allows the precise calculation of the cross-sectional area of muscles, was applied for the segmentation of magnetic resonance images. The choice of technique depended on the morphology of the gluteal muscles and the piriformis muscle.

Gluteal muscles and the piriformis muscle are characterized by heterogeneous fat infiltration and its close proximity, making it difficult to distinguish anatomical boundaries between them. Other segmentation techniques such as automatic segmentation and their algorithms require further improvement to successfully segment these muscles. Additionally, manual segmentation appeared to be the most appropriate method in cases of abundant infiltration of adipose tissue especially relevant in gluteal muscles. Therefore, manual segmentation method appeared to be the most precise method for determining their volume, serving as the gold standard for segmentation [10,15,24]. Additionally, manual segmentation is a conventional method for obtaining CSA [10,11,12,13,14]. The cross-sectional area measurements allow the quantitative assessment of muscle function, which is related to muscle force generation [10,11,12,13,14].

Image segmentation was performed using the ITK-SNAP 4.0 software. Two independent operators served as raters. Areas of four pelvic muscles were segmented: gluteus minimus (bilaterally), gluteus medius (bilaterally), gluteus maximus (bilaterally), piriformis muscle. Muscle tissue was subjected to segmentation, excluding infiltrated fatty tissue within the muscle. Segmentation of the gluteal muscles and piriformis muscle was carried out from the origin of each muscle with clear delineation of muscle borders and filling the delineated area with the assigned color. Raters established standardized methods to create muscle outlines for fat tissue infiltration. The calculations were based on active tissue, excluding subcutaneous adipose tissue (SAT) and intermuscular adipose tissue (IMAT). The line was drawn along the deep fascial plane of each muscle to differentiate SAT from IMAT. Structures were outlined in the manual mode using a traditional mouse creating closed contours of muscles which in the next step were pinpointed on desired area. Those manually created points were connected by the segmentation program. Each segmented muscle was assigned a color for better visibility.

The cross-sectional area measurements require identifying the optimal measurement level. The parameter was measured in mm^3^ in the transverse plane. The CSA of the piriformis muscle was measured at the level of the S4–S5 sacral segments. The CSA of the gluteal muscles was measured bilaterally at the level:

- Of one-third between the iliac crest and the greater trochanter for the smaller and medium-sized gluteal muscles;
- Between the lower and upper part of the acetabulum for the gluteus maximus muscles.

### 2.4. Statistical Analysis

The results were analysed using R software version 4.3.1 and PQStat Software (version 1.8.4.164, 2022) for additional analyses. The analysis included measurements of muscle cross-sectional area (CSA) from two independent raters. To assess the normality of the data, the Shapiro–Wilk test was conducted. Since the data did not follow a normal distribution, non-parametric tests were used and the median and interquartile range were reported. The Mann–Whitney test was performed to assess significant differences between the sides of the muscles, considering the independent nature of the samples. To analyse differences in nominal data, a binomial test for proportions (side to side comparison) and chi-square test (disc prolapse, root compression, sensory deficits, laseque sign and tendon reflex between chronic LBP patients and healthy volunteers) were employed. The significance level was set at 5%.

To evaluate the reliability of cross-sectional area measurements between the two raters, the concordance correlation coefficient and intra-class correlation coefficient were calculated. These coefficients were used to assess the agreement between the raters for the continuous variable of CSA.

## 3. Results

The exact binomial test showed statistically significant muscle atrophy both for left and right pain side for the gluteus maximus, gluteus minimums and piriformis muscle in over 50% of patients with chronic low back pain (Table 2). Moreover, Table 2 showed gluteus medius atrophy in patients with left side pain occurrence (*p* < 0.001). Furthermore, the decrease in CSA for all muscles on the left side can be indicated (Table 2).

The CSA measurements showed significant muscle atrophy (*p*-value < 0.05) in GMax (only confirmed by one rater *p* = 0.049), GMed, GMin and Pir on the symptomatic pain side (Table 3).

In the group of healthy volunteers, there were no statistically significant differences in decrease in the cross-sectional area between right and left sides except GMed (Table 4).

The concordance and intra-class correlations between CSA measurements made by two independent raters in chronic LBP patients and healthy volunteers showed a great inter-rater agreement for all measurement (Table 5). The ICC values were greater than 0.95.

**Figure 1:**
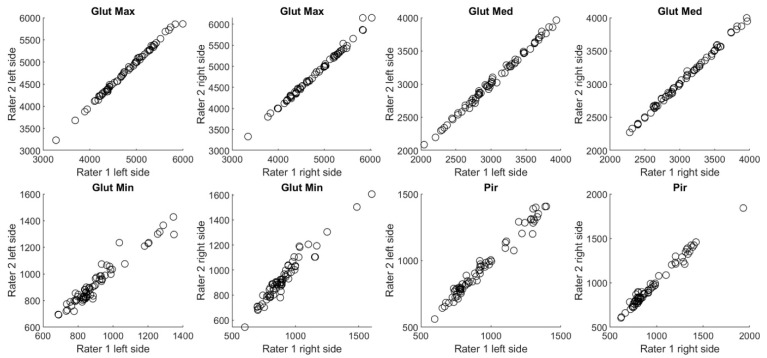
Overall scatter of CSA measurements in [cm^3^] performed by two independent raters (1 and 2) in chronic LBP patients.

**Figure 2:**
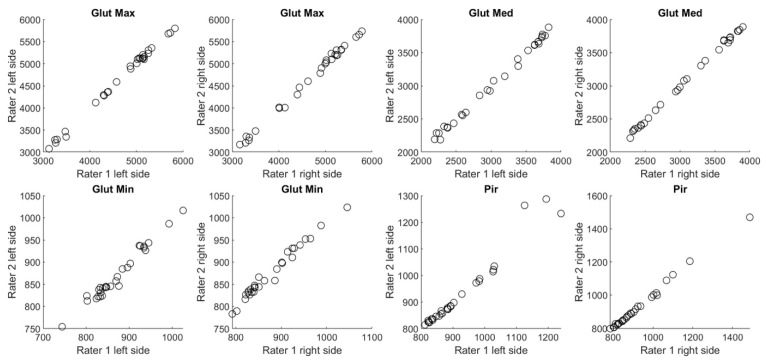
Overall scatter of CSA measurements in [cm^3^] performed by two independent raters (1 and 2) in healthy volunteers.

Figure 1 shows overall scatter of the data relating to the inter-rater reproducibility for each muscle in chronic LBP patients. A 45° degree angle line revealed a good distribution, meaning high inter-rater agreement. The same distribution tendency was confirmed for healthy volunteers (Figure 2).

## 4. Discussion

The comparative analysis of the cross-sectional area of four pelvic muscles showed statistically significant symptomatic atrophy of the gluteus maximus (except rater one assessment), gluteus medius, gluteus minimums and piriformis muscle in more than half of patients with symptoms of chronic low back pain. Moreover, differences in a smaller cross-sectional area indicating muscle atrophy between left and right muscles on the symptomatic side occurred in all inspected muscles, except gluteus medius. In the control group, there were no statistically significant muscle atrophy differences between right and left lower limb in the tested muscles except GMed. Moreover, the concordance correlation coefficient and intra-class correlation coefficient presented great inter-rater reproducibility for each muscle both in patients and healthy volunteers.

For many years, it was assumed that clinically significant morphometric muscle changes related to volume and CSA alterations in patients with chronic LBP occurs in the lumbar spine (mainly in the multifidus muscle) [13,14,15,16]. In the last decade, studies also emerged indicating the utility of assessing pelvic muscles [11]. The theoretical foundations were based on pain syndromes with clinical symptoms resembling sciatica, but with the pathomechanism dependent on changes in the soft tissues of the sacroiliac joint, i.e., sacroiliac joint syndrome, gluteal syndrome and piriformis syndrome. So far, this relationship has been confirmed in only two studies evaluating pelvic muscle atrophy in patients with chronic LBP. The usefulness of applying cross-sectional area measurement of computed tomography images was confirmed presenting the atrophy of gluteus maximus [25]. Similar results concerning GMax were obtained in the conducted study. In one study, no significant atrophy of gluteus maximus was measured but it might be due to the study methodology where the mean values of patients and control group were compared rather than juxtaposing painful to asymptomatic side of the patients as in our analysis [26]. The latest study utilizing MRI segmentation and pelvic muscle volume analysis confirmed statistical importance of atrophy in the gluteus maximus, gluteus minimus and piriformis muscle in over 50% of patients with low back leg pain [10], which is consistent with the results we obtained. The study also showed no statistical importance of gluteus medius atrophy and reported a decrease in ICC values for this muscle. In this research, where ICC were higher, mean CSA values revealed significant muscle atrophy of GMed on the symptomatic pain side. Gluteus medius presented also statistical importance in healthy volunteers, and we observed a tendency for the higher muscle values to occur mainly on the right side. Perhaps limb dominance influenced this result, as gluteus medius plays an important role as a postural muscle and is responsible for maintaining the pelvis stability and lower extremity function [27,28].

Although, volumetric parameter showed a promising method for the indicated muscle atrophy analysis, most studies regarding muscle segmentation relevant to low back pain focus on cross-sectional area measurement [10,11,12,13,14]. Therefore, in this study, patients were assessed with the use of this method. Based on our analysis, we established a statistically significant difference in the cross-sectional area (CSA) for the gluteus maximus, gluteus medius, gluteus minimus and piriformis muscles when comparing the asymptomatic side with the symptomatic side. Although the literature indicated a few limitations of the CSA method, it allows for much quicker analysis and shows good results as volumetric measurement [10,11,12,13,14]. Thus, we can indicate that cross-sectional area parameter might be the appropriate one for pelvic muscle atrophy assessment.

Many studies confirming the validity of magnetic resonance image segmentation application of muscle atrophy symptomatic assessment were conducted in patients with LBP [13,14,15,16]. Most of them focused on the psoas major and multifidus muscle [18,19,21,29,30,31]. Moreover, the relationship of low back pain for the decrease in the cross-sectional area of the psoas major muscle and the increase in adipose tissue infiltration was confirmed [32]. To accurately assess active muscle tissue, this aspect was taken into consideration and, in this study, the muscle CSA measurement did not include adipose tissue infiltration. Based on the meta-analysis of changes in the morphology and composition of the lumbar muscles in patients with low back pain, the cross-sectional area analysis revealed a slight atrophy of the multifidus muscles, but with a significant amount of intramuscular fat infiltration [29]. In addition, the CSA measurements of the paraspinal muscle showed muscle degeneration in patients with degenerative diseases of the lumbar section [33].

Atrophy of the lumbopelvic muscles in patients with chronic LBP has been documented in many cases, but it is not known whether it is the cause or the effect of pain in this area [34]. In the conducted study, tested muscles, except GMax in rater one assessment, showed decreased cross-sectional area for symptomatic side comparing to asymptomatic side. One cause of this state may be reduced physical activity caused by pain. Muscular atrophy on the symptomatic side can be explained by the patient sparing the lower limb to which the pain radiates [11]. Another reason may be secondary atrophy caused by trauma, infections and inflammatory diseases. Another common mechanism of muscle atrophy in patients with LBP is neurogenic atrophy caused by nerve damage due to nerve compression indirectly affecting muscle degeneration on the pain side [34].

In the conducted research, the value of magnetic resonance image segmentation in patients with chronic LBP should be assessed. Magnetic resonance enables the measurement of morphology and composition of the anatomical structures, including the assessment of muscle tissue such as: size, volume and fat infiltration in muscles [10,11,12,13,14]. Although precise standardized protocols and guidelines for the lumbar and pelvic muscles have not been described so far, MRI segmentation seems to be the most appropriate method for muscle atrophy measurements. For this reason, the study used medical image acquisition using magnetic resonance imaging. Moreover, lately, the diagnostic possibility of magnetic resonance segmentation has been indicated to classify patients with chronic low back pain, including sciatica, into clinical subgroups [11,34]. In the conducted study, concordance and interrater measurement was taken into consideration. It showed a high degree of compatibility between raters for each muscle both in patients and healthy volunteers. Therefore, we can assume that the most precise method for cross-sectional area and volumetric measurements remains manual segmentation [10,15,24]. It is also necessary to follow the dynamically developing methods of automatic segmentation, which may contribute to shortening the segmentation time and enable wider diagnostic methods for patients with symptoms of chronic low back pain.

Muscle degeneration in chronic low back pain patients is mainly characterized by decrease in the CSA and increase in fat infiltration [30]. Apart from the muscle atrophy, the usefulness of the muscle fat assessment is also indicated. Perhaps further studies should examine the fat content of the pelvic muscles, which may give more unambiguous results than muscle atrophy measurement. So far, in patients with chronic low back pain, even with small changes in muscle size, a significant amount of intramuscular fat infiltration for the multifidus muscle [29] and the spinal extensor [30] has been proven. Since, in the conducted study, all patients with chronic low back pain showed muscle atrophy compared to healthy volunteers, it should be determined whether the reduced values are not due to an increase in the muscle fat component causing a decrease in active muscle tissue.

Limitations of the Study

There were several limitations in the conducted research. Firstly, there are only a few studies with little data available of gluteal and piriformis muscle atrophy in patients with chronic LBP. Secondly, the available data refer to outdated mechanisms of pain understanding. Moreover, there is no standardized measurement of pelvic muscle atrophy. Then, the main limitations of manual segmentation include the time-consuming process and rather subjective results, which may appear different if performed by another user. Another important issue is the size of the research sample, which should be increased in subsequent studies to increase study reliability. Moreover, it is said that cross-sectional area assessment is a faster but less accurate method of evaluating muscle atrophy than volumetric analysis; thus, the volumetric parameter for pelvic muscle assessment on the same patients should be investigated. Another issue to consider is whether the participants have undergone prior rehabilitation and whether the used rehabilitation methods could potentially impact the analysis results. Additionally, the study did not assess the influence of the duration of symptoms and/or the presence of recurrent low back pain, which could also potentially affect the study outcome.

## 5. Conclusions

The cross-sectional area served as a reliable indicator, showcasing muscle atrophy on the symptomatic side in contrast to the asymptomatic side in chronic low back pain patients. Nonetheless, it is crucial to emphasize that the diagnostic and clinical value of cross-sectional area warrants further investigation through future studies.

## Figures and Tables

**Table 1:** Clinical description of the analyzed group.

Participants	MRI [%]						Neurological Examination [%]							
	Disc Prolapse			Root Compresion			Sensory Deficits				Laseque’s Sign		Tendon Reflex	
													Patellar	Ankle
	L3–4	L4–5	L5–S1	L4	L5	S1	L3	L4	L5	S1	>45	<45	Ascence	Absence
Chronic LBP	26.67	63.33	53.33	60.00	25.00	23.33	16.67	18.33	28.33	23.33	58.33	41.67	5.00	25.00
Healthy volunteers	13.78	44.83	41.38	17.24	20.69	6.90.	0	0	0	0	-	-	3.45	6.90
*p*-value *	0.173	0.101	0.279	0.030	0.624	0.064	-	-	-	-	-	-	0.653	0.053

* *p*—the Chi2.

**Table 2:** Differences in the occurrence of decrease in the cross-sectional area for the left muscles compared to the right muscles in chronic low back pain patients.

Muscle	Painful Side	Rater	*n* = (CSAleft < CSAright)	*n* = (CSAright < CSAleft)	*p*-Value	95% Confidence Interval
GMax	right	1	3	27	<0.001 *	(0, 0.24)
		2	4	26	<0.001 *	(0, 0.28)
	left	1	39	2	<0.001 *	(0.85, 1)
		2	38	3	<0.001 *	(0.82, 1)
GMed	right	1	11	19	0.1002	(0, 0.53)
		2	11	19	0.1002	(0, 0.53)
	left	1	33	8	0.0001 *	(0.68, 1)
		2	33	8	0.0001 *	(0.68, 1)
GMin	right	1	5	25	0.0002 *	(0, 0.32)
		2	6	24	0.0007 *	(0, 0.36)
	left	1	28	13	0.0138 *	(0.54, 1)
		2	30	11	0.0022 *	(0.60, 1)
Pir	right	1	9	21	0.0214 *	(0, 0.47)
		2	9	21	0.0214 *	(0, 0.47)
	left	1	32	9	0.0002 *	(0.65, 1)
		2	32	9	0.0002 *	(0.65, 1)

Legend: * *p* < 0.05; CSAleft—cross-sectional area of left muscle; CSAright—cross-sectional area of right muscle; GMax—Gluteus Maximus; GMed—Gluteus Medius; GMin—Gluteus Minimus; Pir—Piriformis.

**Table 3:** Comparison of the mean cross-sectional area values of the four pelvic muscles (symptomatic vs. asymptomatic side of a group of patients with chronic low back pain).

Muscle	Rater	MV M(IQR)		*p*-Value
		Symptomatic Side	Asymtomatic Side	
GMax	1	4682,39 (4274,82–5121,50)	4845,78 (4424,54–5344,40)	0.056
	2	4669,15 (4289,28–5133,12)	4837,75 (4421,27–5339,89)	0.049 *
GMed	1	2939,9 (2868,07–3274,08)	3097,265 (2843,08–3474,76)	0.030 *
	2	2969,92 (2679,19–3262,24)	3106,24 (2870,69–3471,58)	0.026 *
GMin	1	841,21 (788,42–885,75)	888,45 (845,21–980,39)	0.002 *
	2	861,16 (803,65–984,58)	904,47 (879,72–1043,52)	0.021 *
Pir	1	811,36 (767,26–1003,72)	922,45 (799,12–1200,43)	0.039 *
	2	805,73 (767,29–994,53)	902,56 (823,88–1227,51)	0.007 *

Legend: * *p* < 0.05; GMax—Gluteus Maximus; GMed—Gluteus Medius; GMin—Gluteus Minimus; Pir—Piriformis; MV M(IQR)—median and interquartile.

**Table 4:** Differences in the occurrence of decrease in the cross-sectional area for the left muscles compared to the right muscles in healthy volunteers.

Muscle	Rater	*n* = (CSAleft < CSAright)	*n* = (CSAright < CSAleft)	*p*-Value	95% Confidence Interval
GMax	1	17	12	0.229	(0.42, 1)
	2	16	13	0.355	(0.38, 1)
GMed	1	21	8	0.012 *	(0.56, 1)
	2	21	8	0.012 *	(0.56, 1)
GMin	1	18	11	0.132	(0.45, 1)
	2	18	11	0.132	(0.45, 1)
Pir	1	14	15	0.229	(0, 0.58)
	2	17	12	0.229	(0.42, 1)

Legend: * *p* < 0.05; CSAleft—cross-sectional area of left muscle; CSAright—cross-sectional area of right muscle; GMax—Gluteus Maximus; GMed—Gluteus Medius; GMin—Gluteus Minimus; Pir—Piriformis.

**Table 5:** Concordance and intra-class correlations between cross-sectional area measurements made by two independent raters (1 and 2) in chronic low back pain patients and healthy volunteers.

Muscle	Side	Chronic LBP Patients		Healthy Volunteers	
		Concordance Correlation	ICC	Concordance Correlation	ICC
GMax	right	0.998	0.999	0.998	0.999
	left	0.996	0.996	0.996	0.997
GMed	right	0.998	0.998	0.998	0.998
	left	0.998	0.998	0.998	0.998
GMin	right	0.955	0.955	0.955	0.955
	left	0.952	0.952	0.952	0.952
Pir	right	0.988	0.988	0.988	0.988
	left	0.989	0.989	0.989	0.989

Legend: GMax—Gluteus Maximus; GMed—Gluteus Medius; GMin—Gluteus Minimus; Pir—Piriformis; ICC, LBP—Low Back Pain.

## Data Availability

The data presented in this study are available on request from the corresponding author.
